# Implications of Cellular Immaturity in Necrosis and Microvascularization in Glioblastomas IDH-Wild-Type

**DOI:** 10.3390/clinpract12060108

**Published:** 2022-12-13

**Authors:** Cristian Ionut Orasanu, Mariana Aschie, Mariana Deacu, Madalina Bosoteanu, Sorin Vamesu, Manuela Enciu, Gabriela Izabela Bălţătescu, Georgeta Camelia Cozaru, Anca Florentina Mitroi, Raluca Ioana Voda

**Affiliations:** 1 Clinical Service of Anatomic Pathology, Departments of Pathology, “Sf. Apostol Andrei” Emergency County Hospital, 900591 Constanta, Romania; 2 Center for Research and Development of the Morphological and Genetic Studies of Malignant Pathology (CEDMOG), “Ovidius” University of Constanta, 900591 Constanta, Romania; 3 Faculty of Medicine, “Ovidius” University of Constanta, 900470 Constanta, Romania; 4 Academy of Medical Sciences of Romania, 030167 Bucharest, Romania; 5 Clinical Service of Anatomic Pathology, Departments of Genetics, “Sf. Apostol Andrei” Emergency County Hospital, 900591 Constanta, Romania

**Keywords:** IDH, glioblastoma, microvascular density, necrosis, nestin

## Abstract

Necrosis and increased microvascular density in glioblastoma IDH-wild-type are the consequence of both hypoxia and cellular immaturity. Our study aimed to identify the main clinical-imaging and morphogenetic risk factors associated with tumor necrosis and microvascular in the prognosis of patient survival. We performed a retrospective study (10 years) in which we identified 39 cases. We used IDH1, Ki-67 and Nestin immunomarkers, as well as CDKN2A by FISH. The data were analyzed using SPSS Statistics. The clinical characterization identified only age over 50 years as a risk factor (HR = 3.127). The presence of the tumor residue, as well as the absence of any therapeutic element from the trimodal treatment, were predictive factors of mortality (HR = 1.024, respectively HR = 7.460). Cellular immaturity quantified by Nestin was associated with reduced overall survival (*p* = 0.007). Increased microvascular density was associated with an increased proliferative index (*p* = 0.009) as well as alterations of the CDKN2A gene (*p* < 0.001). CDKN2A deletions and cellular immaturity were associated with an increased percentage of necrosis (*p* < 0.001, respectively, *p* = 0.017). The main risk factors involved in the unfavorable prognosis are moderate and increased Nestin immunointensity, as well as the association of increased microvascular density with age over 50 years. Necrosis was not a risk factor.

## 1. Introduction

Currently, glioblastoma IDH-wild type is a distinct tumor entity from central nervous system gliomas [1]. Two of the criteria that define its diagnosis are tumor necrosis and microvascular proliferation. Other aspects that can be associated with them are represented by TERT mutations, chromosome 10 deletion, trisomy 7, or EGFR amplification [2].

In the case of glioblastomas, necrosis remains an unsolved subject for which several hypotheses have been proposed. The main microscopic aspect is circumscribed necrosis, whose elementary cause is the hypoxic process [3,4].

One of the hypotheses supports the development of necrosis on the same pathogenic pathways of gliomagenesis, PI3k-AKT, and MAPK [5]. Stimulation of AKT and MAPK activities inhibits cell apoptosis, stimulating cell growth with overconsumption of nutrients, ultimately leading to a necrotic process [5,6]. To stimulate these pathways, an increased concentration of glutamate is needed, a concentration that becomes toxic. The cascade of events is followed by the increased intracellular accumulation of cystine, which affects glutathione production, decreasing the neutralization of reactive oxygen radicals. It results in an increased accumulation of reactive oxygen radicals that will lead to the excretion of intracellular calcium. The consequences consist of a massive decrease in ATP and membrane oxidation that will lead to necrosis [6].

Another hypothesis was born through observational studies in which it was noted that areas of necrosis appear in areas where proliferation is intense (increased Ki-67 index) and there is an increased population of pluripotent stem cells (Nestin, SOX,2 and REST positive) [7]. In this case, the necrosis is produced by an imbalance of the increased demand for nutrients by cells with proliferation capacity (immature) and a reduced supply of vascularization [3,7]. Thus, this hypoxic process determines another consequence: angiogenesis, which in the case of these tumors, creates an extensive picture of microvascularization [8].

Over time, four microvascular architectures have been identified: microvascular sprouting, vascular cluster, vascular garland, and glomeruloid vascular proliferation [9]. The first pattern resembles physiological angiogenesis, and its presence denotes a higher survival rate. The vascular cluster is defined by at least two vessels without interposed stroma. The vascular garland is arranged perinecrotically and consists of neoformation vessels with a circular arrangement [9,10]. The glomeruloid proliferation consists of at least two vessels embedded in the stroma, mimicking the appearance of the glomerulus [9,11]. The last two types are associated with an increased proliferation rate and implicitly a bad prognosis [9].

Currently, in the case of glioblastoma IDH-wildtype, only three negative prognostic factors are known: age over 65 years, extensive necrosis, and MGMT promoter methylation status (with the amendment that its major role lies in predicting the therapeutic response) [2]. The therapeutic management of patients consists of surgical resection followed by radiotherapy (focal radiotherapy with doses of 60 Gray) and chemotherapy (temozolomide) [12].

The purpose of this study is to observe the prognostic role in patient survival of the degree of cellular maturity responsible for the hypoxic processes that lead to the appearance of necrosis and microvascularization in a group of patients diagnosed within our service, observing their associations with clinical-imaging parameters and morphogenetics.

## 2. Materials and Methods

We conducted a retrospective study between 1 January 2011 and 31 December 2020 of patients diagnosed with a primary tumor of the central nervous system in the Constanta County Emergency Clinical Hospital, Dobrogea region. The inclusion criteria consisted of adult patients and histopathological diagnosis of grade 4 glioma, IDH-wildtype. The exclusion criteria consisted of necrotic-diagnosed cases and recurrences.

**Figure 1:**
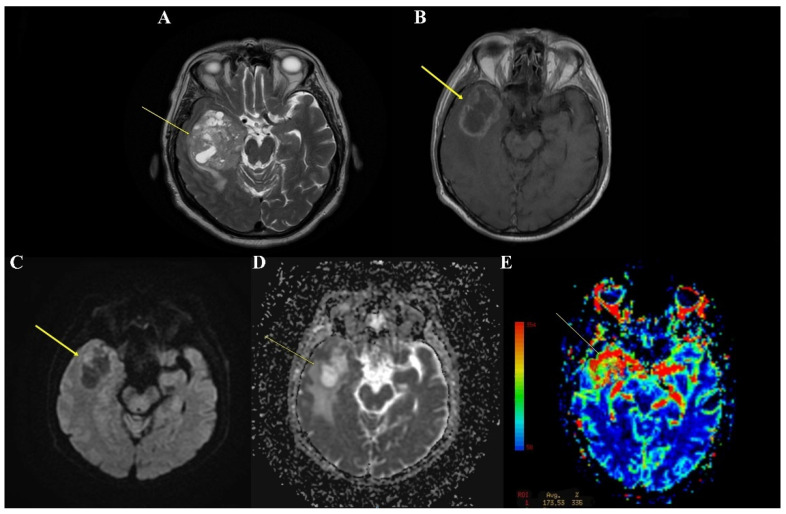
(**A**) MRI axial T2 weighted image reveals a right temporal lobe mass, with a solid/cystic aspect, with maximum axial dimensions of 65/59 mm, surrounded by edema. (**B**) MRI axial T1-weighted with contrast highlights a right temporal lesion with peripheral contrast enhancement. (**C**,**D**) MRI axial DWI and ADC sequences show diffuse restriction in the anterior aspect of the lesion. (**E**) MRI axial PWI shows an increase in rCBV at the lesion level.

The clinical and evolutionary information of the patients came from the hospitalization form. The imaging examination performed before the surgical intervention was done according to the criteria suggested by Nobel J.M. et al. [13]. Location, tumor size, tumor volume, peritumoral edema, and midline shift were monitored (Figure 1). The type of resection was assessed postoperatively by magnetic resonance imaging (MRI).

Sampled tissue was macroscopically described and prepared according to international protocols within the Clinical Service of Anatomic Pathology, County Emergency Hospital, Constanta. Histopathological diagnosis was given by two pathologists according to the latest WHO criteria (2021 edition). The degree of necrosis was assessed as a percentage on all slides stained with hematoxylin-eosin of the respective case, the necrotic volume being calculated from the total tumor volume.

**Figure 2:**
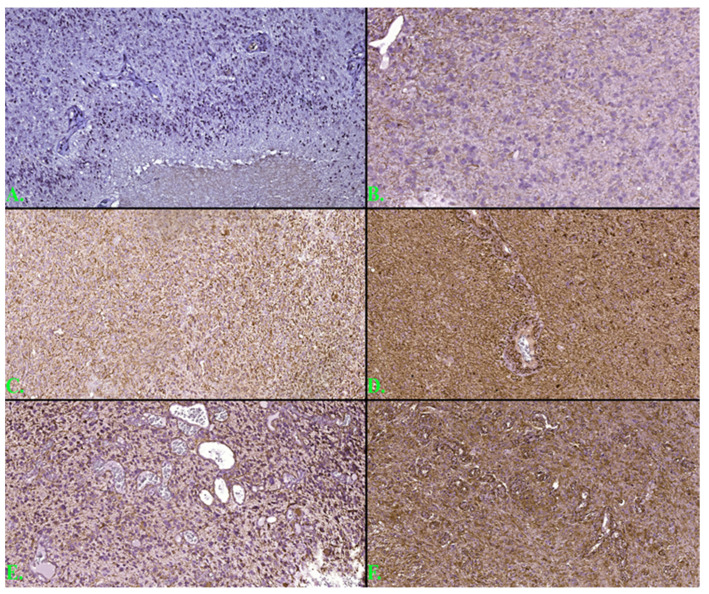
(**A**) The cytoplasmic reaction of IDH in the studied group (ob 100×). (**B**) Weak cytoplasmic immunointensity for the Nestin marker in glial cells (ob 100×). (**C**) Moderate cytoplasmic immunointensity for the Nestin marker in glial cells (ob 100×). (**D**) Strong cytoplasmic immunointensity for the Nestin marker in glial cells (ob 100×). (**E**) Hotspot of microvascular density (45 blood vessels) on a background of moderate cytoplasmic Nestin immunointensity at the cellular level (ob 100×). (**F**) Hotspot of microvascular density (30 blood vessels) on a background of strong Nestin immunointensity at the cellular level (ob 100×).

The immunohistochemical examinations were performed at the Center for Research and Development of the Morphological and Genetic Studies of Malignant Pathology (CEDMOG). Formalin-fixed paraffin-embedded were sectioned at 4 µm and prepared according to the working protocol provided by the manufacturer, Master Diagnostica (Sevilla, Spanish). Immunohistochemical tests were performed by the HIER-DAB method and used the markers IDH1 R132H (H09), Nestin (10C2), and Ki-67 (SP6). The counter-staining was performed with Hematoxylin-Eosin. The IDH1 marker was examined according to reactivity (positive or negative) and the cytoplasmic intensity of the reaction (strong, moderate, or weak). The Nestin marker was evaluated by the cytoplasmic immunoreaction (strong, moderate, or weak) of the glial cells and reactivity (positive or negative) in endothelial cells. Slides were scanned with a TissueScope LE120 Slide Scanner (Huron Digital Pathology, Ontario, CA), and captures were made of 10 microvascular hotspot areas with dimensions of 1 mm^2^ each. The total number of capillary vessels identified by two pathologists was divided by 10, resulting in the average number of vessels per 1 mm^2^. For Ki-67, the reference index was calculated as the percentage of positive nuclei after counting on 10 HPF of at least 1000 nuclei (Figure 2).

The study of the CDKN2A gene was carried out using fluorescent in situ hybridization (FISH) within CEDMOG. The formalin-fixed paraffin-embedded samples were sectioned at 3 µm. The tissue slides were performed according to the manufacturers’ recommendations through pretreatment, denaturation, hybridization, and post-hybridization steps. The cytogenetic evaluation used ZytoLight SPEC CDKN2A/CEN 9 Dual Color Probe probes (Bremerhaven, Germany). Fluorescent signals of the preparations were calculated in 100 tumor nuclei using a fluorescence microscope Zeiss Axio Imager 2 (Zeiss Gmbh, Oberkochen, Germany). In cells without abnormalities, two green (CDKN2A gene region) and two orange signals (CEN 9 probe) were seen. In cells with deletion, fewer orange signals were seen, and in the case of amplification, more orange signals were seen.

Statistical analysis was performed in SPSS Statistics Version 26 (IBM Corporation, New York, NY, USA). Data association was performed using Pearson Correlation Coefficient. Analysis of data was performed by Fisher’s exact test and Chi-square test for categorical data and Mann-Whitney U Test and Kruskal-Wallis H Test for continuous variables. Indicators of central tendency and variability were used. Survival estimates were made until 1 November 2022 and were calculated using the Kaplan-Meier method applying the log-rank test to observe the difference between groups. Hazard ratios (HR) were appreciated by using Cox regression analysis. All results were considered statistically significant at a *p*-value of <0.05.

All patients included in the study signed the informed consent, and the ethics opinion was obtained from the local ethics committee.

## 3. Results

### 3.1. Demographic and Clinical Characteristics of the Patient Group

**Figure 3:**
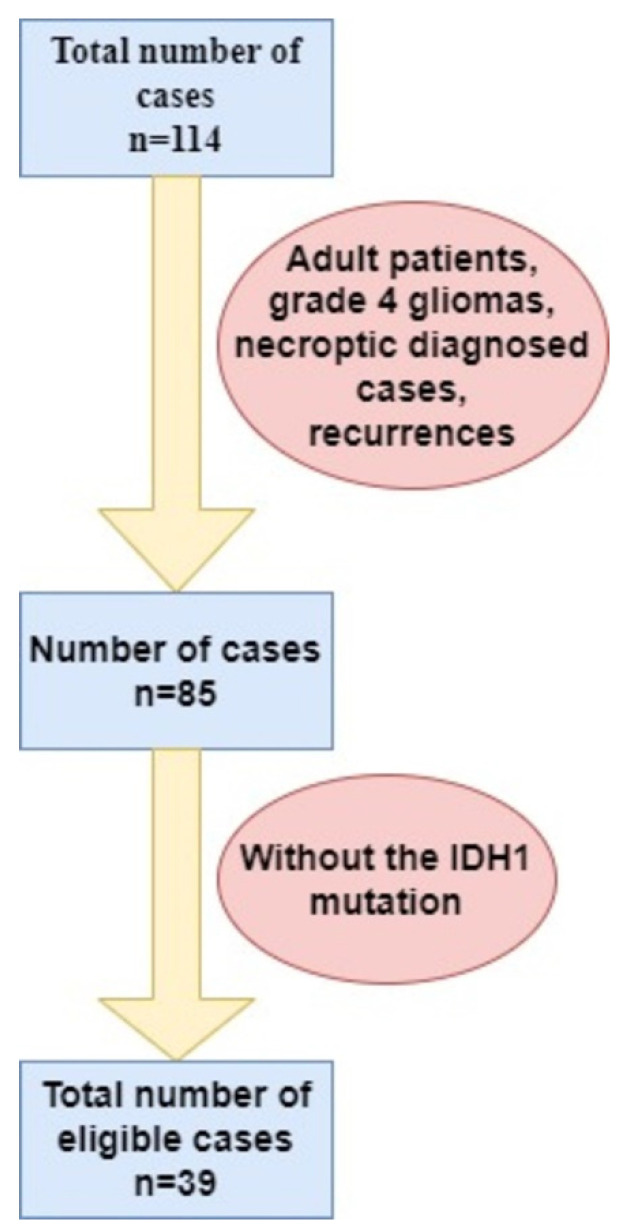
Stratification of cases according to inclusion and exclusion criteria. IDH1—Isocitrate dehydrogenase 1.

In our institution, we identified 114 glial tumors from which, after applying the inclusion and exclusion criteria, 39 IDH-wildtype Glioblastomas were eligible (Figure 3).

**Figure 4:**
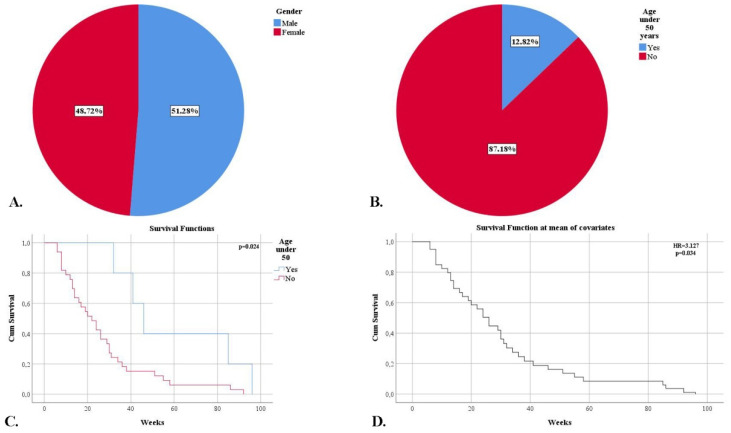
(**A**) Frequency of cases according to gender. (**B**) The frequency of cases after and before the age of 50. (**C**) Kaplan-Meier survival graphic showing a lower survival rate for patients over 50 years of age. (**D**) Univariate cox regression analysis demonstrating mortality risk for patients over 50 years of age.

We noticed a slight male predominance of 51.28%. The average age at diagnosis was 60.36 years (Figure 4A). We observed an increased frequency of cases in the sixth decade of life (38.46%), and 87.18% of patients were over 50 years old (Figure 4B). Moreover, patients under the age of 50 had longer survival (60 weeks versus 26.78 weeks) (*p* = 0.024). Age at the time of diagnosis was an independent risk factor for mortality (HR = 1.047, *p* = 0.017), and the 50-year threshold was also an independent risk factor for mortality (HR = 3.127, *p* = 0.034) (Figure 4C,D).

In 82.05% of cases, the symptomatology had a rapid onset, with a presentation to the doctor in the first month. The most common symptoms were: cognitive disorders (58.97%), headache (58.97%), and paresis (56.41%). No statistically significant correlation was observed between the clinical picture and the demographic aspects. The most common associated comorbidities were hypertension (41.03%) and diabetes (25.64%). They did not present any statistical significance regarding survival.

### 3.2. Imaging Characteristics of the Studied Cases

Most lesions were located supratentorial (94.87%), with two cases located at the level of the cerebellum. The most affected cerebral lobe was the temporal (20.51%), followed by the parietal (17.95%). 97.44% of the lesions had a diameter over 2.5 cm, the average being 5.21 cm. The presence of increased axial diameters was associated with the occurrence of paresis (*p* = 0.034). A statistically significant correlation was observed between tumor sizes and the onset of symptoms. A sudden onset (less than a week after presentation) was associated with lesions exceeding 5 cm (*p* = 0.043 Fisher). The average tumor volume was 91.69 cm^3^. Both the increased dimensions and an increased volume were associated with the presence of headache (*p* = 0.002, respectively, *p* < 0.001). The lesions produced an average midline displacement of 8.51 mm.

The post-interventional imaging documentation observed a complete ablation in 23.08% of cases. The presence of tumor residue was associated with the age of patients over 50 years (*p* = 0.036). The survival rate in the case of total resection was 36.56 weeks in contrast to incomplete resection, 29.48 weeks, but without significant statistical correlation (*p* = 0.595). In the cases of partial resection, the average rate of excised tissue was 82.19%. The resection rate was inversely correlated with age (*p* = 0.006). This was influenced by the amplitude of the midline shift. Thus a larger resection was performed in cases where the structures were greatly deviated (*p* = 0.026). The average residual volume was 14.92 cm^3^. In the case of patients who presented symptoms of intracranial hypertension, an increased presence of the residual volume was observed (*p* = 0.025). Also, the residual volume was directly proportionally associated with both the axial diameter of the tumor and the volume of the tumor (*p* = 0.002, respectively, *p* < 0.001). The presence of an increased residual volume represented an independent risk factor for mortality (HR = 1.024, *p* = 0.013).

After the complete and/or partial surgical intervention, 79.49% of the patients benefited from complete radio-chemotherapeutic treatment (60 Gy in 5 days out of 7, 2 Gy per session in combination with Temodal 75 mg/m^2^ for 42 days, followed by monotherapy 150 mg/m^2^ for 5 days). The trimodal treatment showed an increased average survival according to Table 1 (*p* = 0.004).

The univariate analysis of the data did not identify an independent risk factor regarding the radiochemotherapy treatment or the type of surgical resection. Instead, the multivariate analysis of the data observed an increased risk factor in the case of the association of the absence of chemoradiotherapy and complete ablation (HR = 7.460, *p* = 0.017) and in the case of the absence of chemoradiotherapy and subtotal ablation, the risk factor was a huge one (HR = 46.016, *p* < 0.001).

### 3.3. Histogenetic Aspects of the Analyzed Cases

The histopathological examination identified 85 grade 4 gliomas in which tumor necrosis, microvascular proliferation, and marked cytonuclear atypia were observed. Of these, only 39 cases did not show reactivity to the IDH marker. Two rare subtypes were also identified: three giant cell glioblastomas and one gliosarcoma.

The proliferative index had an average of 38.15%. Increased values of Ki-67 were identified in the case of patients who complained of cognitive disorders (*p* = 0.027). The proliferative rate was not an independent predictor of mortality (*p* = 0.849).

**Figure 5:**
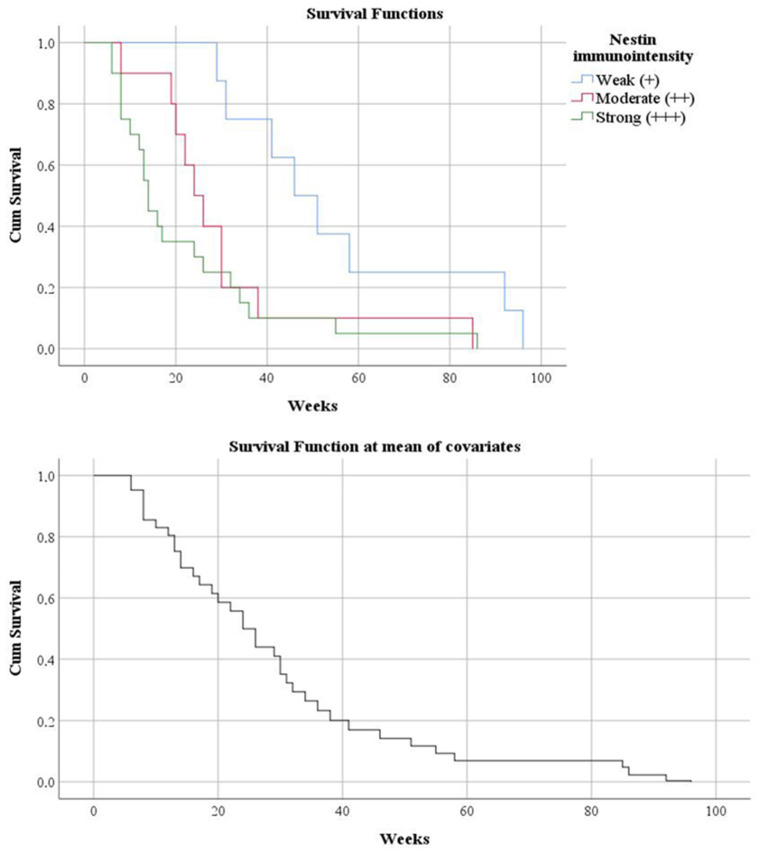
Kaplan-Meier survival graphic (*p* = 0.007) and univariate Cox regression analysis (HR = 4.028, *p* = 0.003, respectively HR = 2.919, *p* = 0.043) show a lower survival rate in stratification by Nestin marker immunointensity.

Regarding the degree of cell maturation achieved with the Nestin marker, we observed a strong immunointensity (immature cells) in 51.28% of cases. An association was observed between the degree of immunointensity and the time elapsed until death; the more immature the cell, the shorter the time (*p* = 0.001). Thus, in cases with cellular immaturity, the average was 21.9 weeks, and in the others, 55.5 weeks (*p* = 0.007). Also, both increased and moderate reactivity represented independent risk factors in predicting mortality (HR = 4.028, *p* = 0.003, respectively HR = 2.919, *p* = 0.043) (Figure 5). In the case of multivariate analysis, it was observed that immature cells and the presence of tumor residue (HR = 2.744, *p* = 0.010), as well as immaturity with acute onset, less than a week and less than a month of symptoms (HR = 4.002, *p* = 0.003, respectively HR = 2.748, *p* = 0.048), were predictive factors of mortality. Also, age below 50 years associated with either moderate or increased immunointensity was a risk factor for death (HR = 3.718, *p* = 0.012, respectively HR = 4.020, *p* = 0.001).

FISH analysis of the CDKN2A gene observed a normal status in 64.10% of cases. An increased proliferative index was associated with gene abnormalities (deletions and amplifications) (*p* = 0.029). Also, gene alterations were associated with a low degree of cell maturity (*p* = 0.012). A higher mortality was observed in the case of deletions (28.72 weeks) and amplifications (16.67 weeks), compared to the normal status of the gene, but without a statistically significant correlation (*p* = 0.250). Univariate and multivariate analyzes did not identify genetic alterations as risk factors for mortality.

The microvascular density (MVD) measured as an average per 1 mm^2^ was 40.39 vessels/mm^2^ (18.60–78.60 vessels/mm^2^). In the case of patients who presented with headaches, the microvascular density was significantly increased (*p* = 0.017). Also, in cases where the number of vessels was increased, a greater presence of residual volume was observed (*p* = 0.033). The proliferative index correlated directly proportionally with MVD (*p* = 0.009). An increase in the number of vessels on the surface was associated with a gradual decrease in cellular maturity (*p* = 0.008). In the case of the CDKN2A gene status, we observed that gene amplifications and deletions show a statistically significant correlation with the angiogenesis process (*p* < 0.001). Univariate analysis did not identify MVD as a risk factor for death (*p* = 0.226). Instead, the multivariate analysis noted the association of MVD either with age over 50 years or with cellular immaturity as risk factors for mortality (HR = 1.019, *p* = 0.011, respectively HR = 1.017, *p* = 0.010).

The average percentage of necrosis identified was 32.26% (5–72%). In the case of patients who presented cognitive disorders, an increased percentage of tumor necrosis was observed (*p* = 0.022). Also, this was strongly and directly proportionally associated with the proliferative index and high microvascular density (*p* < 0.001, respectively *p* < 0.001). In cases where the cellularity showed a gradual decrease in maturity, numerous areas of tumor necrosis were observed (*p* = 0.017). In deletions and amplifications of the CDKN2A gene, an increased necrotic percentage was observed (*p* < 0.001). Univariate and multivariate analyzes did not identify necrosis as a negative risk factor for survival.

## 4. Discussion

Glioblastoma IDH-wild type represents 48.3% of malignant tumors of the central nervous system, with an incidence of 3–4 cases per 100,000 inhabitants [12,14]. It occurs more frequently in the male sex [14]. The average age of diagnosis is 65 years, with an increase in the Nordic countries up to 69–70 years [14,15]. Our study is similar to the data present in the literature, except for the lower average age. The meta-analysis performed by Kim et al. observed a heterogeneity of the data regarding the threshold age in which the risk groups of the patients were divided. Some studies, like ours, have identified age over 50 years as a risk factor for mortality, while in other studies, either a different age cut-off was observed or the fact that age over 50 years did not represent a predictive factor of mortality [15]. Similarly to our study, Barz et al. identified age at presentation as an independent risk factor for mortality (HR = 1.047 in our study and HR = 1.045 in Barz et al.) [16].

In our study, most of the patients went to the doctor in the first month, while in the study conducted by Ohgaki et al., the presentation to the doctor was late. This can also explain the average survival time of the patients in their case, 20.43 weeks (4.7 months), compared to our study, 31.16 weeks (7.17 months) [17]. The symptomatology is not evocative; it depends on the size of the tumor, the peritumoral edema, and its location [18]. Aspects are also identified in our work. The most frequent complaints are represented by headaches, motor deficits, cognitive deficits, and less often epileptic seizures, they being more frequent in low-grade gliomas [18]. As in the study carried out by Pantoja Cavalcante et al. and in our case, the most frequent symptoms were represented by headache and motor deficits (paresis) [19].

Several studies, including ours, have highlighted the fact that the average preoperative tumor volume is not a predictive factor of mortality; it correlates with age, symptoms, or the methylation status of the MGMT promoter [16,20,21]. Instead, the residual volume and, implicitly, the incomplete resection represent a risk factor for mortality, respectively determining a low survival [16,22]. Our research identified the presence of postoperative tumor volume as a risk factor for mortality (HR = 1.024), a result similar to the study conducted by Bette S et al. (HR = 1.036) [20].

Over time, optimization and identification of the most reliable prognostic factor in terms of survival have been attempted. Various studies have noted a greater survival in the case of a residual volume of less than 2 cm^3^ or less than 5 cm^3^ associated with minimal resection of 95% or 70% [23,24]. In the case of recurrences, an 80% resection would improve survival [25]. Other studies have identified the fact that the residual volume is more important in patient survival than the type of resection, and the focus should fall on its quantification [26,27,28]. In this sense, the surgical approach technique must involve new techniques such as fluorescent guidance, brain mapping techniques, intraoperative radiotherapy, optical coherence tomography, etc. [22].

The current standard treatment of patients with glioblastoma provides maximum surgical resection, radiotherapy, and the administration of temozolomide (even if, for various reasons, there is resistance to this) [29]. However, in our study, it was highlighted that the absence of any element of this trimodal treatment represented a risk factor for mortality and implicitly decreased survival (Table 1). To reduce the overburdening of the health system, the hypothesis related to the delay of radiochemotherapy was circulated, with no differences observed between its early administration or after a time interval. However, this varies depending on other considerations, such as the histopathological subtype, the type of resection, or DNA methylation [30].

The wild-type status of the IDH gene gives high-grade gliomas a bad prognosis and an increased proliferative rate [31]. In our study, despite an increased proliferative rate, it was not associated with an increase in mortality, not representing an independent or associated risk factor regarding death. The studies carried out by Armocida D et al., and Dumke R et al. highlighted both the fact that an index greater than 20% predicts a low survival and the fact that a larger lesion shows increased proliferation [32,33]. However, Ki-67 cannot be validated as a prognostic factor because it shows intra- and interobserver variability; it depends on the tumor heterogeneity as well as the examined area [32,34,35].

In our study, the proliferative index was statistically significantly associated with CDKN2A gene deletions and amplifications. Located on chromosome 9, this gene plays a role in the pathogenesis of various types of gliomas, even low-grade ones [36]. Gene alterations, especially homozygous deletions, are associated with a poor prognosis and a low survival rate in the case of IDH-mutant gliomas [31,36]. However, in the case of IDH-wildtype gliomas, it does not represent an independent prognostic factor of mortality [37]. This aspect was also identified in our study. Even if we observed a low survival rate in the case of genetic abnormalities (but without a statistically significant correlation), its role in survival and as a prognostic factor cannot be affirmed. Several studies have managed to highlight the importance of the CDKN2A gene only in association with the MGMT methylation promoter status. In this case, only the CDKN2A homozygous deletion and MGMT unmethylated status represent a negative prognostic factor [31,38].

The heterogeneity of IDH-wild-type gliomas has opened the way for researchers to identify the most important biomolecular characteristics to treat and increase survival. Most studies have focused on the tumor immuno-microenvironment [39]. However, two topics that should not be neglected are represented by microvascularization density and tumor necrosis. It is well known that in the case of IDH-mutant gliomas, extensive necrosis and microvascularization are prognostic factors. In the case of the wild-type status of the IDH gene, these aspects remain only histopathological characteristics [2]. Therefore, our study tried to identify their impact in conjunction with cellular immaturity in the prognosis and survival of patients.

The alterations that appear in the pathogenesis of glioblastoma are based on a population of immature cells (stem-like cells) with increased proliferative potential [29,40]. The increased replicative potential and the immature phenotype are the consequence of hypoxia [41]. Hypoxia is a phenomenon self-maintained by the angiogenesis process. This process creates a system of tumor vascularization with low amounts of oxygen and nutrients but enough for immature cells to proliferate. Foci of necrosis are also caused by hypoxia, and the pseudo-palisade appearance is a characteristic. Poor diffusion in oxygen causes cell death forming necrotic centers. The immature cells in the respective area migrate to a better-oxygenated area, forming this palisade arrangement around the necrosis and proliferation [42].

Nestin is a major component of the cytoskeleton and is included in type VI of intermediate filaments [43]. It is involved in the organization of the cytoskeleton, cell signaling, organogenesis, and cell metabolism and has an antiapoptotic function by inhibiting caspase activation [44,45]. It can be expressed in many tissues such as bones, brain, marrow, lung, gastrointestinal tract, etc. [43]. At the level of the normal and mature central nervous system, it can be located in a few cells arranged in the ventricular wall and the subependymal tissue [44]. Its activity decreases with cell differentiation, being replaced by other intermediate filaments (GFAP) so that the expression is functionally associated with a stem cell [7,43,46]. It is expressed in neural stem cells, cancer stem cells, and undifferentiated cancer cells [44]. In some tumors, such as cerebral ones, its overexpression correlates with tumor grade and invasiveness and indicates cellular immaturity [46]; it gives cells an aggressive character manifested by its characteristics: cell self-renewal, proliferation, survival, differentiation, and migration (metastases and/or dissemination) [43,45].

These characteristics of aggressiveness were also found in our study, observing the fact that in cases where the cells were intensely positive for Nestin, they were associated with a reduced time to death. We also identified the fact that moderate and high degrees of cellular immaturity/differentiation represent important risk factors in terms of mortality. Thus, we can associate the importance of its role in the prognosis of glioblastomas IDH-wild type with that of the CDKN2A gene in IDH-mutant gliomas. Other aspects identified, which would strengthen its importance as a marker of prognosis, consist of the associations between cellular immaturity with the acute onset of symptoms, with age over 50 years, and with the presence of tumor residue.

Nestin is also useful in the evaluation of newly formed blood vessels. Its expression was detected in endothelial cells of embryonic capillaries and endothelial progenitor cells [47]. A particular aspect is the fact that in mature endothelial cells, immunoreactivity is negative [47,48]. This angiogenesis of gliomas initially started from the hypothesis of the migration of mesenchymal stem cells of the hematogenous marrow [49]. Subsequent studies in vivo and in vitro showed that the process originates from the transdifferentiation of progenitor glial cells or neural stem cells [50]. The evaluation of the angiogenesis process and, implicitly, the density of microvascularization by Nestin is similar to other specific vascular markers such as CD105—Endoglin, CD31, and CD34 [51,52,53]. The most important advantage that Nestin confers, compared to the other immunomarkers, is represented by the fact that it reacts only with neoformation vessels, not with mature vessels or normal tissue [54,55].

Regardless of the quantification method or the immunomarker used, the microvascular density in grade 4 gliomas is high; an aspect also evoked in our data [54,56,57,58]. As in the study conducted by Tena-Suck ML et al., the microvascular density quantified by us was associated with symptoms (epilepsy in their case), cellular immaturity, and an increased proliferative index [59]. A particularity identified by us is represented by the association between CDKN2A gene alterations and high MVD in IDH-wild-type cases. This aspect was not found in the specialized literature; the only study identified that analyzes these two parameters were carried out by Appay et al. They evaluated in IDH-mutant cases, CDKN2A gene alterations (homozygous deletions) and MVD, not observing associations between the two [36]. Meta-analysis performed by Fan C. et al. noted that a higher MVD was associated with low patient survival. But the data analyzed by them either compares low-grade gliomas with high-grade ones or between different socio-demographic factors such as ethnicity [60]. Until now, there have been no studies focused only on IDH-wild-type glioblastoma due to its heterogeneity. Even if, in our data, MVD does not represent an independent factor of death, the multivariate analysis found that the association with age over 50 years or with cellular immaturity has a low prognosis on patient survival.

In addition to the angiogenesis process, hypoxia is also responsible for the presence of necrosis [59]. The self-maintaining relationship between cellular immaturity, cellular proliferation, and MVD was also highlighted in our study by the associations of the percentage of necrosis with these parameters [41,42]. Besides these, we noted the association of necrosis with the altered status of the CDKN2A gene, an aspect also noted by other researchers but in cases of IDH-mutant gliomas [36,61]. Even if the technique was different (imaging vs. histopathological), the results of the study conducted by Palpan Flores A. et al. were similar to ours; no significant statistical correlations were identified between the tumor necrosis ratio and survival [62].

The limitations of this study are represented by a small number of cases and the study of microvascularization and cellular immaturity by immunohistochemical method. More studies in this field should investigate these aspects also by immunofluorescence or Western blot. Instead, the strengths are represented by the study of an extremely heterogeneous tumor (glioblastoma IDH-wild type), with few identified prognostic factors in a clinical-imaging-morphogenetic manner that can be the basis of future studies on larger groups in order to the identification of some infallible prognostic factors as was achieved in the case of astrocytoma grade 4 IDH-mutant. Also, the study exposes the bivalence of an immunomarker (Nestin) that must be included in the standard analysis kit of this tumor entity because it provides information important about the degree of maturity of the cell population and about intratumoral microvascularization, both aspects having a role in the prognosis of patients and the possibility of developing a targeted treatment.

## 5. Conclusions

Our study managed to evaluate the clinicalimaging and morphogenetic parameters in correlation with the aspects of cellular immaturity in glioblastoma IDH-wild-type maintained by the MVD and necrosis. The most important aspects regarding the negative prognosis in patient survival were represented by advanced age, the presence of residual volume, and the absence of at least one therapeutic method from the standard trimodal treatment.

The presence of a major immature cell population with moderate and increased immunointensities in Nestin represented an independent risk factor for death. In addition to this aspect, immaturity combined with the acute onset (less than 1 month) of symptoms or age over 50 years or with the presence of tumor residue represented major negative risk factors in terms of patient survival. The percentage quantification of necrosis did not represent a risk factor, while associations of microvascular density with age over 50 years and cellular immaturity represented risk factors for mortality.

## Figures and Tables

**Table 1:** Average survival according to treatment.

Means for Survival Time						
After the surgical intervention	Radiotherapy	Chemotherapy	Mean			*p*-value
			Weeks	C.I. 95%		
	No	No	7.71	6.692	8.737	*p* = 0.004
		Yes	26.00	26.000	26.000	
	Yes	No	12.00	12.000	12.000	
		Yes	37.65	28.730	46.581	

## Data Availability

Not applicable.
